# Differentiating benign from malignant gallbladder wall thickening in non-contrast MRI imaging: Preliminary study of a combined diagnostic indicator

**DOI:** 10.1097/MD.0000000000030861

**Published:** 2022-10-07

**Authors:** Wen-Wen He, Jian-Guo Zhu, Dmytro Pylypenko, Fei Liu, Mei Wang, Yue-Fei Wu, Jun Tian, Hai-Ge Li

**Affiliations:** a From the Department of Radiology, The Second Affiliated Hospital of Nanjing Medical University, Nanjing, Jiangsu Province, P.R. China; b From the GE Healthcare, Beijing, P.R. China.

**Keywords:** carcinoma, diagnosis, DWI, gallbladder, layered pattern, MRI

## Abstract

To synthetically evaluate the diagnostic accuracy of image features for differentiating benign from malignant gallbladder wall thickening disease with non-contrast MRI and establish the optimal diagnostic indicator. A total of 23 patients with wall thickening type gallbladder carcinoma and 61 patients with benign wall thickening disease were included. The diagnostic performance of six image features including the layered pattern on T2-weighted imaging (T2WI) and diffusion-weighted imaging (DWI) images, T2WI signal intensity, papillary growth, the apparent diffusion coefficient (ADC) value, and the lesion to liver parenchyma ratio (LLR) of gallbladder were evaluated and compared. The receiver operating characteristic (ROC) curve and binary logistic regression analysis were used to construct the optimally combined indicator. All six indicators showed high diagnostic accuracy. The layered pattern on DWI and LLR had the highest area under the curve (AUC) value (0.904), followed by the layered pattern on T2WI (0.883), T2WI signal intensity (0.859), ADC value (0.836), and papillary growth (0.796). There was no statistically significant difference in the AUC among indicators for pairwise comparisons. A combination of layered patterns on DWI and papillary growth was shown to be the optimal indicator by binary logistic regression analysis. The AUC value of the combination (0.972) was higher than the layered pattern on DWI (0.904) and papillary growth (0.796) (*P* < .001). Non-contrast MRI provides several reliable indicators for differentiating benign from malignant gallbladder thickening disease. The combination of layered patterns on DWI and papillary growth is the optimal indicator.

## 1. Introduction

Gallbladder wall (GB) thickening is a common radiological manifestation in benign and malignant diseases and frequently leads to diagnostic dilemmas.^[1]^ Common etiologies of GB thickening are acute and chronic cholecystitis, adenomyomatosis, adenomatous Polyps, xanthogranulomatous cholecystitis (XGC), and gallbladder carcinoma (GBC). GBC accounts for 98% of all GB malignancies.^[2]^ GB lymphoma, metastasis, and immunoglobulin G4 related sclerosing cholecystitis are rare causes of GB wall thickening.^[1,3]^ On the other hand, systemic diseases that lead to GB edema, such as congestive heart failure, renal failure, hepatitis, liver cirrhosis, hypoalbuminemia, and inflammation spread from adjacent organs, are common extrinsic causes.^[4,5]^

GBC ranks the sixth among gastrointestinal cancers. It has a low occurrence of <2 cases per population of 100,000 worldwide and has marked ethnic and geographical variations.^[6,7]^ Approximately 80% of GBCs involved in the liver were invasive when they were diagnosed, since the GB subserosa is weak, and there is no peritoneal covering on the contacting surface with the liver.^[8]^ Because of the insidious onset, rapid progression, and advanced stage at diagnosis, GBC has a poor prognosis with an overall 5-year survival rate of less than 5%. However, a 5-year survival rate of 75% can be reached if stage-adjusted therapy is performed in the early stages.^[9]^ Therefore, early diagnosis of GBC is extremely important. At the early stage of GBC, the thickened wall may be the only detectable imaging sign.^[10]^

MRI is now widely used in diagnosing GB disease, particularly in resolving diagnostic difficulties, because of its excellent tissue contrast and high spatial resolution. Enhanced MRI is known for potentially offering additional information about the lesion. However, it is not a routine investigation, and manifestations of some early GBC cases that are similar to benign diseases (e.g., acute and chronic cholecystitis) may prevent further advanced examination. In recent years, there have been controversial discussions regarding the correlation between gadolinium-based contrast agents and nephrogenic systemic fibrosis, and the risk of gadolinium-accumulation in the brain. Nevertheless, precise conclusions have not been drawn yet.^[11–13]^ Therefore, it is worthwhile to explore the diagnostic value of non-contrast MRI examination, which provides shorter scan time, lower costs, less patient inconvenience, and fewer health concerns for patients worried about the use of gadolinium.

As the imaging feature of wall thickening type GBC overlaps with benign lesions, it is challenging to make a differential diagnosis. Non-contrast MRI is an excellent imaging modality for differentiating benign from malignant GB wall thickening. Several useful diagnostic indicators including traditional imaging features, layered patterns of GB wall thickening, and apparent diffusion coefficient (ADC) value measurement have been confirmed to have high diagnostic accuracy for wall thickening type GBC.^[14–16]^ However, few previous studies have attempted to perform a comprehensive comparison of the diagnostic accuracy of these indicators. In addition, no research to our knowledge has been focused on constructing a combined diagnosis indicator that may improve diagnostic accuracy. Currently, multi-parameter combined analysis has been widely used in various aspects of biomedical studies, particularly in the increasing application of radiomics in tumors,^[17–20]^ but as far as we know, this has not been previously explored in the diagnoses of wall thickening type GBC with non-contrast MRI.

Therefore, we hypothesize that there are differences in these image features of wall thickening type GBC in terms of diagnostic accuracy, and an optimal diagnostic indicator can be constructed.

Therefore, we hypothesize that there are differences in these image features of wall thickening type GBC in terms of diagnostic accuracy, and an optimal diagnostic indicator can be constructed.

## 2. Methods

### 2.1. Subjects

**Figure 1.**
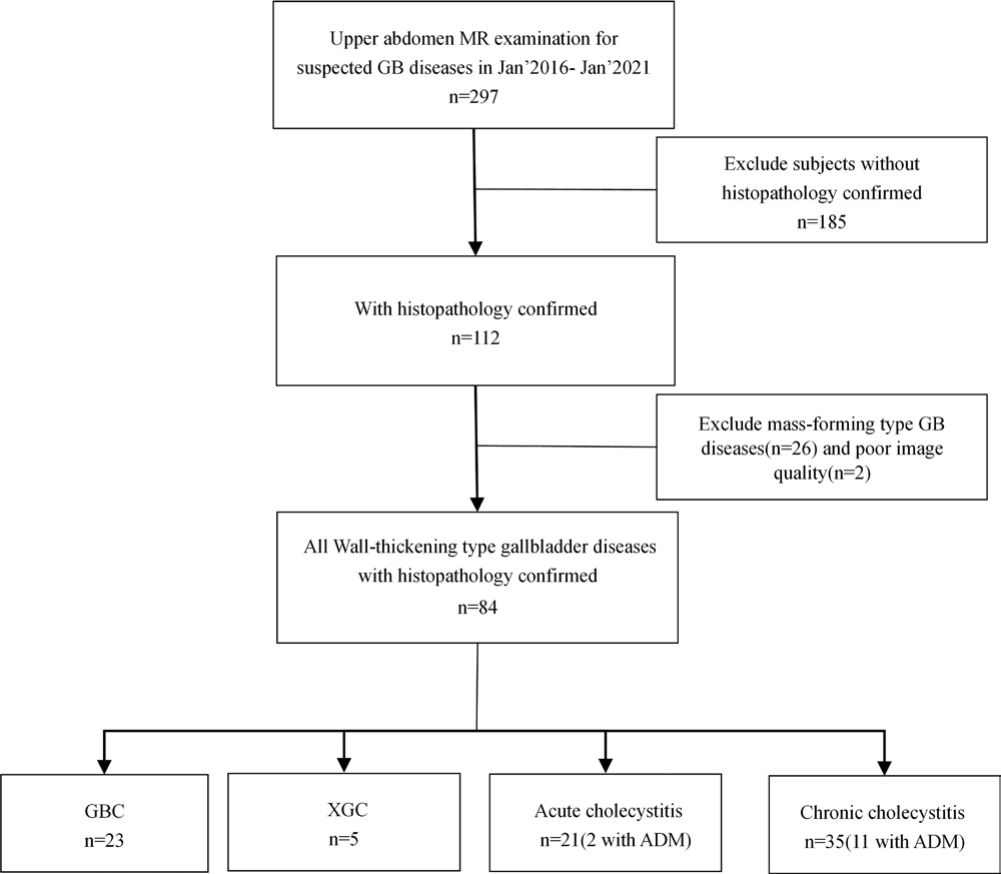
Flowchart of inclusion and exclusion criteria for subjects with wall-thickening type gallbladder diseases. ADM = adenomyomatosis, GB = gallbladder carcinoma, MR = magnetic resonance, XGC = xanthogranulomatous cholecystitis.

This retrospective study was approved by the Hospital Ethics Committee (approval number 2018XJ014). The requirement for informed consent was waived due to the retrospective nature of the study. By retrieving medical records and MRI reports, we collected 297 cases that had performed upper abdomen MRI scans for suspected GB diseases in the last 5 years (2016.1–2021.1) at our hospital. Among them, 185 cases were excluded due to the absence of pathological confirmation, 26 cases were excluded due to the presence of mass-forming type GB disease, and 2 cases were excluded due to poor image quality. Inclusion criteria: patients with suspected GB diseases who had complete MR image and pathological data; wall-thickening type gallbladder lesions (gallbladder wall thickening more than 3mm, with mass-like lesions excluded); image quality was fit for evaluation: gallbladder lesions and their surrounding structures could be clearly displayed without artifacts. Figure 1 illustrates the details of the inclusion and exclusion process. T2-weighted imaging (T2WI) and diffusion-weighted imaging (DWI) images of all cases were then evaluated. GB wall thickening was considered when wall thickness ≥3 mm. The study population consisted of 44 males and 40 females. The average age was 60.32 ± 1.32 years old. All the lesions were obtained with histological confirmation, including 21 cases of acute cholecystitis (2 cases of gangrenous cholecystitis and 1 case of acute suppurative cholecystitis), 35 cases of chronic cholecystitis, 5 cases of XGC, 23 cases of GBC, and 13 cases of adenomyomatosis accompanied with acute and chronic cholecystitis. GBCs included 19 cases of moderately to poorly differentiated adenocarcinoma, 2 cases of well-differentiated adenocarcinoma, and 2 cases of tubular adenomas with high-grade intraepithelial neoplasia and localized cancerous lesion.

MR scans were performed on 3.0-T (HDxt, GE Medical System, Milwaukee) with 8-channel body coils. Patients were asked to fast for a minimum of 4 hour. The MR scan sequences included axial T2-weighted fast spin-echo with fat suppression, liver acquisition with acceleration volume acquisition, DWI, coronal fast imaging employing steady-state acquisition, and three-dimensional MR cholangiopancreatography.

DWI was performed using a single-shot spin-echo-planar imaging sequence by applying diffusion gradients in 3 orthogonal directions for each section, with 2 diffusion weightings (b = 0 s/mm^2^, 800 s/mm^2^). The detailed parameters of the MRI sequences are shown in Table 1.

**Table 1:** MRI sequences and parameters.

Sequence	TR/TE (msec)	ETL	FA	Bandwidth (Hz/pixel)	Slice thickness/spacing (mm)	FOV (cm)	Matrix	No. of excitations
LAVA	6.12/3.13	1	12°	166.67	5.00/0.00	42	512 × 512	1
Fs-T2WI	8000.00/86.02	18	90°	83.33	6.00/2.00	40	512 × 512	2
FIESTA	3.43/1.48	1	65°	125	7.00/1.00	35	512 × 512	1
MRCP	3333.34/800.50	70	90°	62.5	1.80/0.00	38	512 × 512	1
DWI	6666-11250/6373	1	90°	62.5–250	4.00/0.00	40	256 × 256	4

All the sequences were performed using respiratory gating.

DWI = diffusion-weighted imaging, ETL = echo train length, FA = flip angle, FOV = field of view, FIESTA = fast imaging employing steady state acquisition, Fs = fat saturation, LAVA = acceleration volume acquisition, MRCP = magnetic resonance cholangiopancreatography, T2WI = T2-wighted imaging, TE = echo time, TR = repetition time.

### 2.2. Image analysis

The studies were retrospectively reviewed by two abdominal radiologists with 10 years of experience, who disregarded the clinicopathologic information. They evaluated the images using PACS workstation independently in random order. Referring to previous studies,^[14–16]^ the following imaging characteristics were selected to be evaluated by two radiologists: presence of papillary configuration on T2WI images; low, moderate, high, and nondetectable recorded signal intensity of the lesions relative to the spleen on T2WI images; the layered pattern on T2WI images; the layered pattern on DWI images. Discrepancies were discussed and resolved by consensus between reviewers.

The layered appearance was defined as inner and outer layers that could be identified on each slice image of the GB. Based on the research of Lee NK et al,^[16]^ GB lesions were classified into 5 types, according to T2WI and DWI images (Table 2). These patterns were categorized as benign (types 1, 2, 5) and malignant group (types 3, 4) on T2WI and DWI images, respectively.

**Table 2:** Classification of layered patterns on T2WI and DWI images for wall-thickening type gallbladder diseases.

Classification	Layered pattern on T2WI	Layered pattern on DWI
Type 1	Thin GB wall without layered manifestation	Regular GB wall without layered manifestation and diffusion restriction
Type 2	Two-layered appearance, with thin hypointense internal layer and thick hyperintense external layer	Two-layered appearance, with regular internal layer and relatively thick external layer without diffusion-restricted
Type 3	localized thickened wall without layered manifestation	localized thickened wall with diffusion restriction and without layered manifestation
Type 4	Diffusely thickened wall without layered manifestation	Diffusely thickened wall with diffusion restriction and without layered manifestation
Type 5	Multiple small-sized cystic spaces regularly arranged in the wall	Multiple small hyperintensity regularly arranged in the wall

DWI = diffusion weighted imaging, GB = gallbladder, T2WI = T2-wighted imaging.

Two other abdominal radiologists with 5 to 10 years of experience quantitatively analyzed the ADC value at Workstation 4.6 (GE Healthcare). ADC values of the lesion and normal liver parenchyma on the same images were quantified by manually drawing circular regions of interest (ROIs) on the ADC map of the DWI. ROIs were measured twice, and the mean ADC was calculated. Intramural abscesses hemorrhages were avoided when possible. Lesion to liver parenchyma ratio (LLR) was defined as the ratio of the mean ADC value of lesion and mean ADC value of normal liver parenchyma.

### 2.3. Statistical analysis

Quantitative data was expressed as mean ± standard deviation (SD) if statistical data conformed to a normal distribution, and the data that did not conform was as median (quartile spacing). Qualitative data was expressed as frequency and constituent ratio (%).

The interobserver reliability was calculated by the Cohen’s Kappa coefficient for categorical data and the intraclass correlation coefficient (ICC) for quantitative data. Cohen’s kappa and ICC results were classified as follows: >0.80, excellent; 0.61 to 0.80, good; 0.41 to 0.60, medium; and <0.4, poor.

The frequency of papillary growth, pattern analysis on T2WI and DWI images were compared between the benign and malignant groups using Fisher’s exact test. The comparison of T2WI signal intensity between the benign and malignant groups was performed using the Mann–Whitney *U* test. An independent two-sample *t* test was used to compare groups for ADC value and LRR if the data met normal distribution and variance homogeneity; otherwise, the Mann–Whitney *U* test was used.

The diagnostic capability of each image feature which had shown a significant difference in the univariate analysis was evaluated using receiver operator characteristic (ROC) curve analysis.

The image features with statistical significance in the univariate analysis were taken into binary logistic regression analysis, using the forward stepwise method to build a final model. Multiple ROC curves were compared using the DeLong test.

Analysis of ROC curves was calculated using MedCalc (version 19.1.2; Mariakerke, Belgium). Other statistical analyses were performed using SPSS (version 26; Chicago, IL). Differences with a *P* value less than .05 were considered statistically significant.

## 3. Results

The mean age of GBC (67.30 ± 10.58 yr) was higher than that of the benign group (57.69 ± 11.69 yr) (*P* < .001). There were no differences in gender between the malignant (13 females and 10 males) and benign group (27 females and 34 males) (*P* > .05). The wall thickness of GBC (median 1.1 cm, interquartile range 0.70 cm) was thicker than that of the benign group (median 0.50 cm, interquartile range 0.50 cm) (*P* < .001).

### 3.1. Interobserver agreement

The agreements were “good” to “excellent” for pattern analysis on T2WI and DWI images, T2 signal intensity, and papillary growth between two reviewers (kappa values were 0.772, 0.862, 0.707, and 0.772, respectively). ICC values for mean ADC value measurements at GB lesions and normal liver parenchyma were “good” to “excellent” (ICCs were 0.849 and 0.797, respectively) between two radiologists.

### 3.2. Evaluation of diagnostic indicators

There were significant differences between benign and malignant groups in the pattern analysis on T2WI and DWI images, T2WI signal intensity, papillary growth (Table 3).

**Table 3:** Non-quantitative MRI image features in two group of subjects with gallbladder carcinoma and benign disease.

MRI feature	GB carcinoma (N = 23)	Benign disease (N = 61)	Total (N = 84)	*P* value
Layered pattern on T2WI Images				<.001
Reviewer 1				
Malignant disease	21 (91.3%)	9 (14.8%)	30 (35.7%)	
Benign disease	2 (8.7%)	52 (85.2%)	54 (64.3%)	
Reviewer 2				
Malignant disease	21 (91.3%)	12 (19.7%)	33 (39.3%)	
Benign disease	2 (8.7%)	49 (80.3%)	51 (60.7%)	
Layered pattern on DWI Images				<.001
Reviewer 1				
Malignant disease	22 (95.7%)	7 (11.3%)	29 (34.5%)	
Benign disease	1 (4.3%)	54 (88.5%)	55 (65.5%)	
Reviewer 2				
Malignant disease	22 (95.7%)	8 (13.1%)	30 (35.7%)	
Benign disease	1 (4.3%)	53 (86.9%)	54 (64.3%)	
Papillary growth				<.001
Reviewer 1				
Present	17 (73.9%)	9 (14.8%)	26 (31.0%)	
Absent	6 (26.1%)	52(85.2%)	58 (69.0%)	
Reviewer 2				
Present	16 (69.6%)	8 (13.1%)	24 (28.6%)	
Absent	7 (30.4%)	53 (86.9%)	60 (71.4%)	
Degree of T2WI hyperintensity				<.001
Reviewer 1				
Low	20 (87.0%)	12 (19.7%)	32 (38.1%)	
Moderate	1 (4.3%)	10 (16.4%)	11 (13.1%)	
High	2 (8.7%)	17 (27.9%)	19 (22.6%)	
No visibility	0 (0.0%)	22 (36.1%)	22 (26.2%)	
Reviewer 2				
Low	17 (73.9%)	9 (14.8%)	26 (30.9%)	
Moderate	5 (21.7%)	8 (13.1%)	13 (15.5%)	
High	1 (4.3%)	22 (36.1%)	23 (27.4%)	
No visibility	0 (0.0%)	22 (36.1%)	22 (26.2%)	

DWI = diffusion weighted imaging, GB = gallbladder, T2WI = T2-wighted imaging.

Among those 84 lesions, 30 were characterized as malignant (21 type 3 lesions and 9 type 4 lesions) and 54 as benign (17 type 1 lesions, 28 type 2 lesions, and 9 type 5 lesions) on T2WI pattern analysis; 29 were categorized as malignant (21 type 3 lesions, 8 type 4 lesions) and 55 as benign (14 type 1 lesions, 37 type 2 lesions, 4 type 5 lesions) on DWI analysis.

Layered pattern on T2WI showed 91.3% sensitivity, 85.2% specificity, 70.0% PPV, and 96.3% NPV; Layered pattern on DWI presented 95.7% sensitivity, 88.5% specificity, 75.6% PPV, and 98.2% NPV; and papillary growth displayed 73.9% sensitivity, 85.2% specificity, 65.4% PPV, 90.0% NPV for diagnosing GBC.

The mean ADC value of GBC was (1.33 ± 0.49) × 10^−3^ mm^2^/second, which was lower than that of the benign lesions ([1.85 ± 0.53] × 10^−3^ mm^2^/second [*P* < .0001]).

The mean LLR of GBC was 1.01, lower than that of the benign lesions (1.50) (*P* < .0001).

### 3.3. Comparison of ROC curves

The area under the curve (AUC) (95% CI) for determining GBC was 0.883 (0.794–0.943) for layered pattern on T2WI, 0.904 (0.821–0.958) for layered pattern on DWI, 0.796 (0.694–0.876) for papillary growth, 0.859 (0.766–0.925) for T2WI signal intensity, 0.836 (0.740–0.908) for ADC value, and 0.904 (0.820–0.957) for LLR.

The cutoff value of ADC value was less than 1.31 × 10^−3^ mm^2^/second, corresponding to the optimal Youden index (J = 0.619), with 78.26% sensitivity and 83.61% specificity.

The cutoff value of LLR was less than 1.19, corresponding to the optimal Youden index (J = 0.749), with 91.30% sensitivity and 83.61% specificity.

**Figure 2.**
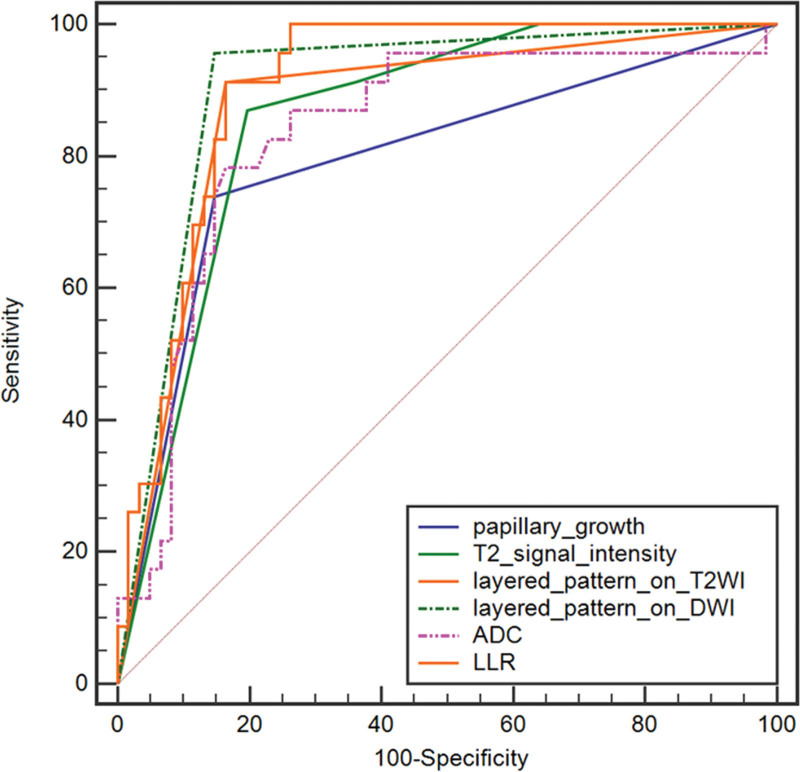
ROC curves of layered pattern on T2WI and DWI images, T2 signal intensity, papillary growth, ADC and LLR. The AUC was 0.883, 0.904, 0.796, 0.859, 0.836, 0.904 for layered pattern on T2WI, layered pattern on DWI, T2 signal intensity, papillary growth, ADC and LLR, respectively. The difference between them did not reach the statistical significance. AUC = area under the curve, DWI = diffusion-weighted imaging, LLR = lesion to liver parenchyma ratio, T2WI = T2-wighted imaging.

There was no statistically significant difference in the AUCs between the layered pattern on T2WI and DWI, T2WI signal intensity, papillary growth, ADC, and LLR. ROC curves of the six indicators mentioned above are shown in Figure 2.

The optimal diagnostic model equation was obtained by binary logistic regression: Logit (P) = −1.539 + 4.612 × layered_pattern_on_DWI + 2.851 × papillary_growth. The layered pattern on DWI and papillary growth were included in the regression model, while T2WI signal intensity, the layered pattern on T2WI, ADC, and LLR were excluded. The ROC curve was fitted through the predictive values of the model.

**Figure 3.**
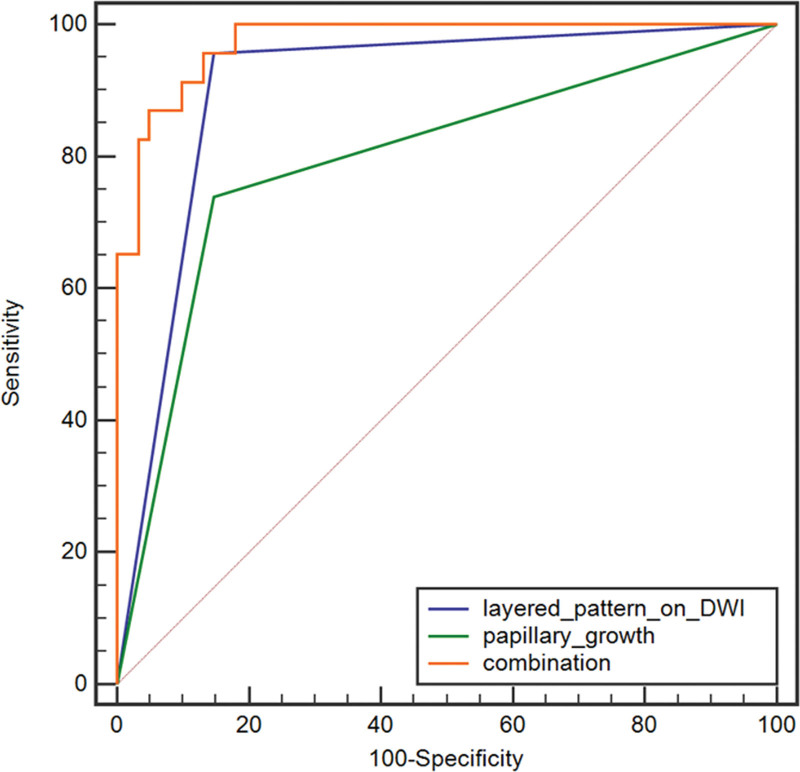
ROC curves of layered pattern on DWI, papillary growth, and the combination of them. The AUC of the combination was 0.972, which was higher than that of layered pattern on DWI and papillary growth alone (*P* < .01). AUC = area under the curve, DWI = diffusion-weighted imaging, ROC = receiver operating characteristic curve.

The AUC value of the combination of the layered pattern on DWI and papillary growth was 0.972 (0.821–0.958), with 95.65% sensitivity and 86.89% specificity, higher than that of each indicator alone (*P* < .01) (Fig. 3).

## 4. Discussion

In this study, the results suggest that all six image indicators (the layered pattern on T2WI and DWI images, T2 signal intensity, papillary growth, ADC, and LLR) were shown to have high diagnostic accuracy. In addition, the layered pattern on DWI combined with papillary growth was demonstrated as the optimal indicator for differentiating benign GB thickening diseases and wall-thickening type GBC in non-contrast MR scans. The results also confirm the usefulness of the quantitative indicator LLR, which reflects the contrast of ADC value between GB lesions and liver parenchyma.

### 4.1. Morphology characteristics

**Figure 4.**
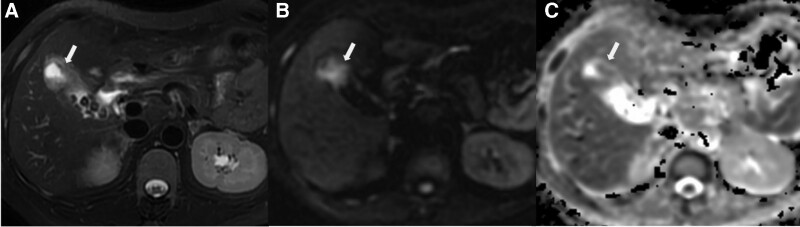
Moderately-poorly differentiated gallbladder adenocarcinoma in a 48-year-old female. (a) Focal wall thickening without layered appearance in the fundus of gallbladder (arrow) was shown on T2WI fat saturation image (type 3), with papillary growth pattern; (b, c) Localized hyperintensity (arrow) was shown on DWI and hypointensity (arrow) on ADC map, without layered pattern (type 3). DWI = diffusion-weighted imaging, T2WI = T2-wighted imaging.

**Figure 5.**
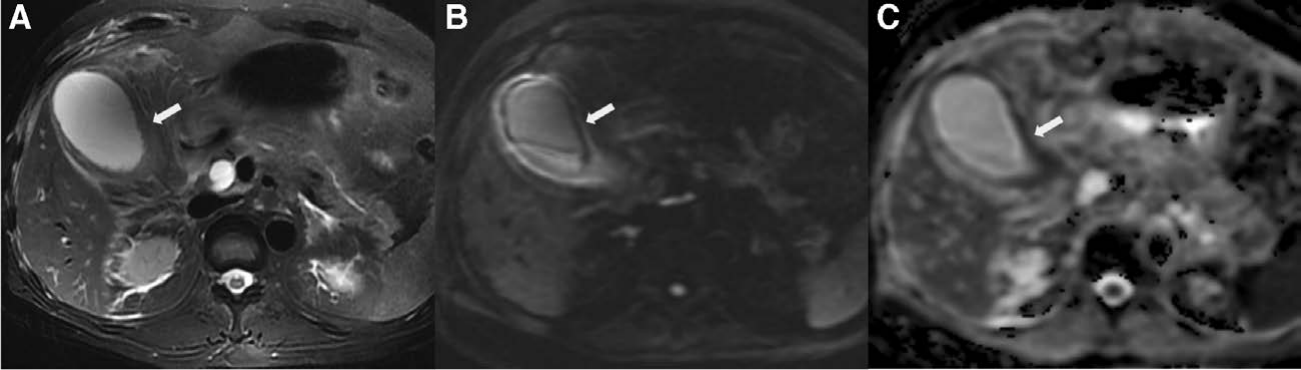
Acute gangrenous cholecystitis in a 49-year-old male. (a) Diffuse hypointensity of thickening (arrow) was exhibited in the thickened GB wall on T2WI with fat saturation image, without layered pattern (type 4); The inner wall appeared somewhat papillary change due to mucosal irregularity; (b) Diffuse hyperintensity with layered pattern on DWI images was shown (arrow); (c) The layered pattern was observed on ADC map image, with hypointensity (arrow) in the neck of GB (type 2). DWI = diffusion-weighted imaging, GB = gallbladder, T2WI = T2-wighted imaging.

The most common patterns of GBC are mass forming and diffuse or focal wall thickening, which represents the infiltrative growth pattern of tumor, with or without irregular thickened wall.^[21,22]^ Papillary configuration is generally considered a malignant tumor feature, it can occur in the early stage of mass forming type tumor, and can be the manifestation of wall thickening type GBC (Fig. 4). However, this feature is not observed clearly in some wall thickening type GBC cases. Additionally, few GB infection cases show analogous papillary changes due to the irregular appearance of the inner wall of GB (Fig. 5). A previous study shows the observed papillary growth pattern to be less than half (41.7%) in mild wall thickening type GBC, while it was 4.6% in benign GB thickening disease.^[14]^ In this study, papillary changes in GBC lesions (73.9%) are observed more than that in the above-mentioned report, as it might include more advanced lesions. It is also observed in 9 (14.8%) begin conditions (1 in XGC, 1 in chronic cholecystitis, 7 in acute cholecystitis). The papillary growth pattern is highly suggestive of GBC (*P* < .001), but it should be differentiated from the mucosal irregularity in inflammatory lesions. Because of some infiltrative growth patterns, GBC lesions may not present a papillary appearance, more attention should be paid to prevent misdiagnosis.

**Figure 6.**
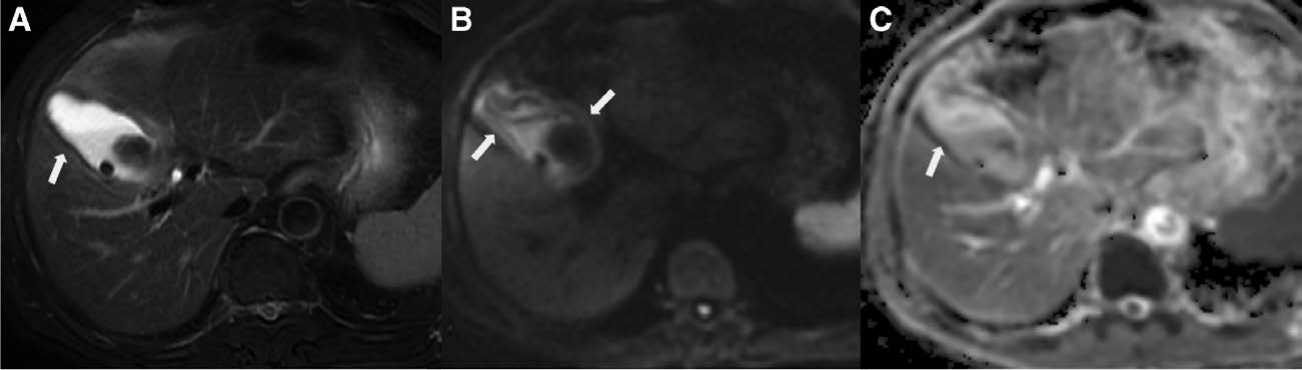
XGC in a 68-year-old male. (a) Diffuse gallbladder wall thickening (arrow) without layered appearance was shown on T2WI with fat saturation image (type 4). Hypointensity in the inner margin of the gallbladder was observed, without papillary growth pattern. (b) Diffuse hyperintensity (arrow) was shown on DWI, with layered appearance. (c) Hypointensity (arrow) was observed in the inner margin on ADC map image. DWI = diffusion-weighted imaging, T2WI = T2-wighted imaging.

On T2WI sequences, GBC lesion is usually hyperintense with some inhomogeneity relative to the liver.^[21]^ Consistent with the data reported by a previous study,^[14]^ the results in this study show that T2 signal intensity could distinguish benign and malignant benign and malignant GB lesions. Compared to the spleen, most GBC lesions (87%) were presented with low or moderate T2WI, while most cholecystitis lesions were observed with high intensity. This may be due to less intra-tumoral water content in GBC than in benign conditions, which consist of non-neoplastic lesions. However, it is very challenging to distinguish GBC from XGC through T2WI signal intensity, because most XGC is observed with low or moderate intensity, as is GBC (Fig. 6).

### 4.2. Layered patterns

**Figure 7.**
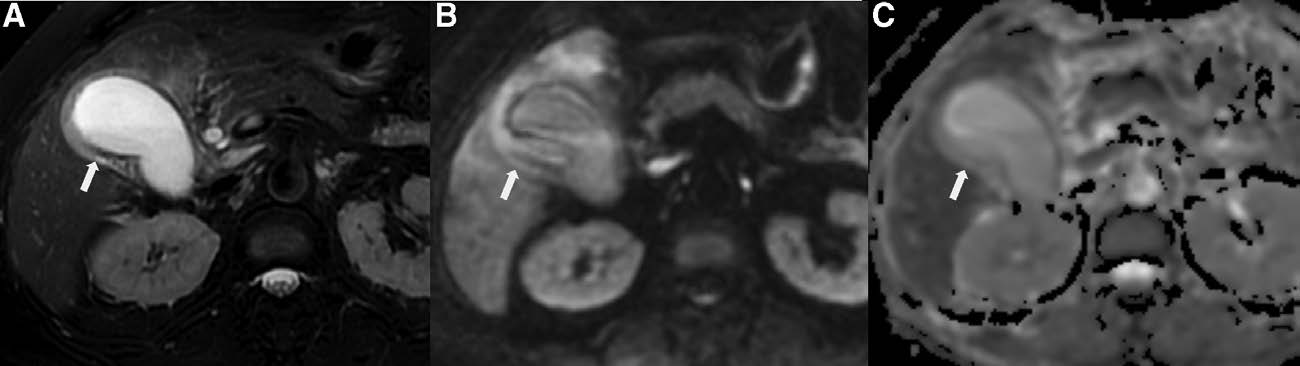
Acute onset of chronic cholecystitis in a 61-year-old female. (a)Diffuse gallbladder wall thickening (arrow) with layered appearance was exhibited on T2WI with fat saturation image (type 2); Hypointensity was observed in the inner wall of GB and hyperintensity in the outer wall, without papillary change. (b) Diffuse hyperintensity with layered appearance on DWI image was shown (arrow). (c)The layered pattern (arrow) was shown more clearly on the ADC map image (type 2). DWI = diffusion-weighted imaging, T2WI = T2-wighted imaging.

Several studies have attempted to distinguish malignant from benign GB wall thickening based on the classification of layered patterns.^[15,16,23]^ In these previous studies, most GBC displayed focal or diffuse wall thickening without layered appearance, which may correspond to the infiltration of the tumor into the wall, while most benign diseases (mainly inflammatory lesion) appeared layered change due to acute inflammatory cell infiltrations or serosal edema^[15]^ (Figs. 4 and 7).

Jung et al demonstrated the correlation between classification of the layered pattern on T2WI images and pathologic findings.^[15]^ The results presented high sensitivity (92%) and specificity (97%) of layered patterns for diagnosing GBC and provided a useful diagnostic marker.

NK. Lee et al highlighted the role of DWI by adding the feature of diffusion restriction for GBC and demonstrated the diagnostic accuracy of layered patterns for GBC on combined T2WI and DWI images (sensitivity, 97.2%; specificity, 92.2%; PPV, 83.3%; NPV, 98.8%) was slightly higher than that on T2WI (sensitivity, 97.2%; specificity, 86.7%; PPV, 74.5%; NPV, 98.7%).^[16]^ In their study, the subjects included not only had wall-thickening type diseases but also mass-forming type lesions.

Although this study had excluded the mass-forming type lesions, the results were still similar to that of the previous study mentioned above. The diagnostic accuracy of layered pattern on DWI was slightly higher than that of layered pattern on T2WI for wall thickening type GBC. However, the difference between the diagnostic performance of T2WI and DWI layered patterns did not reach statistical significance. In addition, the combined DWI layered patterns and papillary growth patterns had a higher predicted probability than T2WI layered patterns and other single indicators.

In conclusion, consistent with previous studies,^[15,16,23]^ the results of this study strongly suggest that the focal or diffuse wall thickening with diffusion restriction and without layered patterns accurately indicate GBC. Layered patterns on DWI provide better diagnostic reliability compared to T2WI.

### 4.3. Quantitative DWI

DWI is widely used to distinguish benign from malignant lesions by ADC values.^[20,24,25]^ ADC values in various malignant lesions generally tend to be decreased, probably due to increased tissue cellularity or cell density in malignancy. Many studies have reported the diagnostic value of ADC value^[13]^ for differentiating GBC and benign GB diseases.^[16,25–27]^ In these studies, the optimal ADC cutoff value varied from 1.20 × 10^−3^ mm^2^/second to 1.64 × 10^−3^ mm^2^/second. In this study, the optimal ADC cutoff value for GBC in this research was similar to previous works.

We also attempted to evaluate the diagnostic accuracy of LLR to decrease deviation and compare the diagnostic accuracy of ADC value and LLR for the diagnosis of GBC. Previous studies either conducted an absolute ADC value assessment or simply a qualitative comparison between benign and malignant GB lesions with normal liver parenchyma.^[14,25,28,29]^

Results of this study show that both ADC value and LLR presented good diagnostic performance for GBC (*P* < .001). The AUC of LLR (0.904, 0.820 to 0.957) was higher than that of the ADC value (0.804, 0.740 to 0.908). Although the difference between ADC value and LLR did not achieve statistical significance, it reveals that the diagnostic capability of LLR is not inferior to ADC absolute value for wall thickening type GBC, and even has the potential to be superior to it.

### 4.4. The optimal diagnostic indicator

Results of the binary logistic regression analysis show that layered patterns on DWI and papillary growth were identified as the optimal diagnostic indicators. The combination of these two indicators improved the AUC value to 0.972, which is higher than that of indicators alone, suggesting that the GB wall thickening without layered changes, exhibiting diffusion restriction on DWI, adding papillary appearance is the most reliable imaging parameter for GBC. Meanwhile, high diagnostic value still exists in other indicators such as T2WI signal intensity, pattern analysis on T2WI, ADC, and LLR, with great AUCs of more than 0.80. Our findings suggest more attention should be paid to the layered pattern on DWI and papillary growth of the lesion when diagnosis challenges were encountered. Papillary appearance, lower, moderate, or darker intensity on T2WI, wall thickening without a layered pattern on T2WI, ADC value of fewer than 1.31 × 10^−3^ mm^2^/second, and LLR of less than 1.19 are also highly suggestive of GBC.

### 4.5. Limitations

Our study had several limitations. Firstly, restricted by pathological and imaging data of the cases, selection bias cannot be avoided. Secondly, subgroup analyses for benign conditions were not performed due to the relatively small case number. Thirdly, we did not carry out a radiomics study, nor did we perform an external validation for our model due to our sample size. Moreover, our study is limited because enhanced MR imaging features were not included in the analysis. More cases will be collected for further analysis and additional validation is required by prospective studies with larger samples in the future.

## 5. Conclusions

In conclusion, the high diagnostic accuracy of non-contrast MRI for wall thickening type GBC is due to the usefulness of several effective indicators, including the layered pattern on T2WI and DWI images, T2WI signal intensity, papillary growth, ADC, and LLR. The layered pattern on DWI combined with papillary growth is conformed as the optimal indicator for differentiating benign from malignant GB wall thickening disease with non-contrast MRI.

## Author contributions

Hai-Ge Li and Jian-Guo Zhu conceived the study, designed the study protocol. Wenwen He wrote the manuscript. Wenwen He, Fei Liu, Mei Wang, Yue-Fei Wu and Jun Tian are in charge of coordination and direct implementation. Wenwen He, Fei Liu, Mei Wang and Yue-Fei Wu helped to develop the study measures and analyses. All authors contributed to drafting this study protocol manuscript and have read and approved the final version.

**Conceptualization:** Wen-Wen He, Hai-Ge Li.

**Data curation:** Wen-Wen He, Fei Liu, Mei Wang, Yue-Fei Wu.

**Formal analysis:** Wen-Wen He, Jian-Guo Zhu.

**Investigation:** Wen-Wen He, Fei Liu, Mei Wang, Yue-Fei Wu, Jun Tian.

**Methodology:** Wen-Wen He, Jian-Guo Zhu.

**Project administration:** Wen-Wen He.

**Supervision:** Hai-Ge Li.

**Writing – original draft:** Wen-Wen He.

**Writing – review & editing:** Jian-Guo Zhu, Dmytro Pylypenko.
